# Effectiveness and safety of fluoroscopy-guided acupuncture for subacromial impingement syndrome: A protocol for a randomized, patient-assessor blind, parallel clinical trial

**DOI:** 10.1097/MD.0000000000012386

**Published:** 2018-09-21

**Authors:** Jihye Seo, Seung-Ryong Yeom, Hee-Ra Shin, Kyungtae Park, Jae Kyoun Kim, Soo-Ji Park, Sangkwan Lee

**Affiliations:** a Clinical Trial Center; b Department of Korean Medicine Rehabilitation, Gwangju Medical Center, College of Korean Medicine, Wonkwang University, Gwangju; c Department of Global Public Health and Korean Medicine Management, Graduate School, Kyung Hee University, Seoul; d Department of Internal Medicine, College of Korean Medicine, Wonkwang University, Iksan, Jeonbuk, Republic of Korea.

**Keywords:** acupuncture, fluoroscopy, randomized clinical trials, subacromial impingement syndrome

## Abstract

**Introduction:**

Subacromial impingement syndrome (SIS) is one of the most common diseases inducing shoulder pain. Acupuncture is a source of pain relief that has been used for SIS; however, there is no clinical study about image-guided acupuncture for SIS. The aim of this study is to evaluate the effectiveness and safety of fluoroscopy-guided acupuncture in patients with SIS.

**Methods:**

This is the protocol for a randomized, patient-assessor blind, parallel clinical trial comparing fluoroscopy-guided acupuncture to acupuncture without fluoroscopy. There will be a 1-week run-in period before random allocation to 2 groups. The total duration of the clinical trial will be 3 weeks including 2 weeks for the follow-up period. A total of 57 participants will be enrolled for the clinical trial. Acupuncture will be conducted in 2 sessions for each group, once at baseline and once at the 1-week mark. The primary outcome will be 100 mm pain visual analogue scale. Secondary outcomes will include the Neer and Hawkins test, the Modified Constant Murley Score scale, the Shoulder Pain And Disability Index, the patient satisfaction degree, and the accuracy rate of acupuncture. For the evaluation of safety, adverse events will be monitored.

**Discussion:**

We designed the clinical trial using image-guided acupuncture. This will be the first trial to study the effectiveness of image-guided acupuncture for SIS compared with acupuncture using the proportional measurements.

**Trial registration:**

Clinical Research Information Service Registration Number is KCT0002751. Registered on March 23, 2018.

## 1 Introduction

Subacromial impingement syndrome (SIS) is one of the most common causes of shoulder pain and can cause rotator cuff tendinitis.^[1,2]^ SIS is caused by narrowing of the subacromial space due to various causes, and it can result in shoulder pain, limitation of movement, and loss of strength.^[3,4]^

Currently, treatments of SIS include medications such as analgesic agents, conservative therapies such as exercise and physical therapy, and injection therapy.^[5]^ If conservative therapies fail, steroid injections are most commonly used as the next line of therapy.^[6]^

Acupuncture is an effective treatment for chronic pain such as back pain, knee pain, lumbar pain, and shoulder pain.^[7]^ For shoulder pain, acupuncture has the effects of pain relief, improvement of range of motion, and improvement of shoulder function, and these effects are sustained for up to 6 months, suggesting a long-term effect.^[8]^ In the previous study about SIS, acupuncture and exercise therapy was shown to be effective in improving shoulder function over 12 months.^[9]^

Because the subacromial space is anatomically narrow, it is difficult to inject into the space accurately.^[10]^ Conventional acupuncture treatment, which determines the depth of acupuncture based on the proportional measurements without the aid of imaging devices, may be more difficult to insert needles into the exact target space.

In this study, we would like to use the portable fluoroscopic X-ray system (PF-X-ray, MX-DRF0815, Nano Focus Ray, Republic of Korea) to guide acupuncture into the subacromial space. Because fluoroscopy can guide the bones better than the ultrasound guide used for soft tissues, it is effective for guiding insertion into the narrow acupoint space between the acromion and the humerus.

We designed this study protocol to evaluate the effects and the safety of fluoroscopy-guided acupuncture for SIS, compared with acupuncture without fluoroscopy.

## 2 Methods and design

### 2.1 Study design and ethics approval

**Figure 1:**
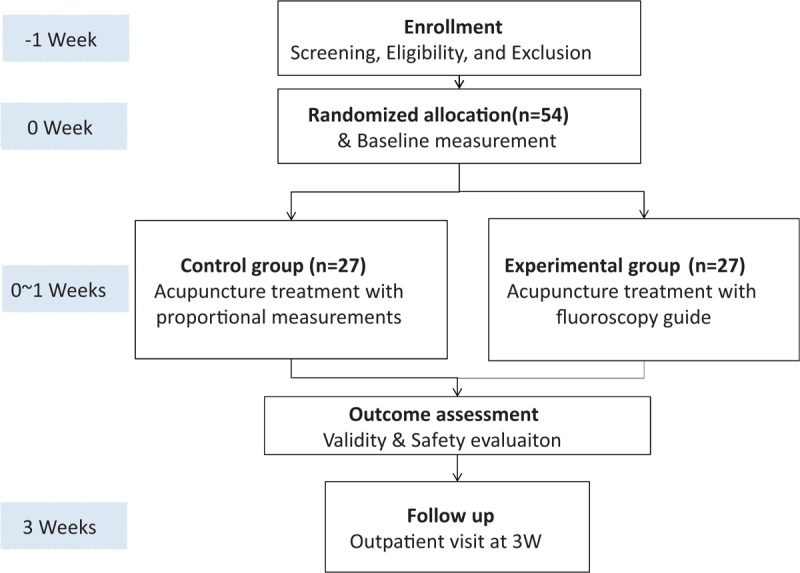
Flow of the study.

This study will be a randomized, patient-assessor blind, controlled clinical trial with 2 parallel groups. The protocol of this study has been approved by the institutional review board (IRB) of Wonkwang University Gwangju Hospital (WKIRB-2017/13) on October 28, 2017. This trial study will be conducted in Wonkwang University Gwangju Hospital in Korea according to the ethical principles of the “Helsinki Declaration.” We will make a poster and advertise it to recruit participants. Before screening for participating in this trial, participants will receive information about the trial. Researchers will proceed with the trial after obtaining a voluntary signature consent, and any identifiable records will be kept confidential. Recruited participants will be randomly assigned to 2 groups: acupuncture treatment with fluoroscopy-guided implementation and acupuncture treatment with proportional measurements. There is a 1-week run-in period before randomization, and total study periods will be 3 weeks, including a 2-week follow-up period. Participants will receive 2 sessions of acupuncture treatment: at baseline (visit 2) and 1 week later (visit 3) (Fig. 1).

### 2.2 Inclusion and exclusion criteria

Inclusion criteria for enrollment are described as follows: males or females aged 25 to 65 years old, with no limitation of range of shoulder motion; those who can be diagnosed as SIS by excluding fracture, dislocation, degenerative arthropathy, os acromiale, subacromial spur, and calcific tendinitis in plain radiography; those with shoulder pain lasting more than 2 months from the onset, the pain is worse when sleeping sideways or lifting the arm over the head, with positive on the Neer or Hawkins test, with more than 50 mm on pain of visual analogue scale (VAS); those who are able to communicate sufficiently with the researcher and complete a questionnaire with minimal help; those who have pledged not to receive treatment other than the prescribed treatment within the period of study; those who agree with written informed consent voluntarily.

Exclusion criteria are described as follows: those who have trauma history of shoulder surgery, fracture, dislocation; those with disorders of the cervical spine or upper extremity that have a significant impact on the shoulder; those who have received injection treatment on the shoulder within the past 6 months; patients with fracture, dislocation, degenerative arthropathy of the glenohumeral joint or acromioclavicular joint, os acromiale, subacromial spur and calcific tendinitis of the shoulder in plain radiography; those with positive findings in a physical examination (drop arm test, empty can test) for exclusion of rotator cuff tear; those with positive findings in the Yergason test for exclusion of biceps tendinitis; those who have been diagnosed with a frozen shoulder (adhesive capsulitis) in a range of motion test; those with pacemaker insertion or significant arrhythmia on electrocardiography); pregnant or breastfeeding females with positive findings in the urine pregnancy test before random assignment, or those who are unable or unwilling to use contraceptive methods (hormonal contraceptives, condoms, intrauterine devices, etc.) to avoid pregnancy throughout the entire study period; those with mental disorders who cannot follow the study protocol; those who were considered as unsuitable for participation by the researcher.

#### 2.2.1 Drop out and early termination

When there is a severe adverse event from the subject or a requirement of suspension, trials can be terminated early, and blinding will be released. In the case of a violation of crucial trial plan or by the researcher's judgement, the subject can be dropped from the study. All data and treatment for the drop out will be recorded in case report form.

#### 2.2.2 Concomitant treatment

A low dose of aspirin (maximum 200 mg per day) for the prevention of stroke is acceptable. Other concomitant treatments that took place 4 weeks or more before enrollment are also acceptable when the researcher judges that the trial results cannot be affected by them.

Any analgesics for pain relief, muscle relaxants, antidepressants, and anticonvulsants are prohibited. However, use of such analgesics can be acceptable if the subject stopped taking the medicine 1 week before the trial. The same standard applies to physical therapy, injection, and treatments performed by other hospitals for pain relief.

### 2.3 Randomization and blinding

Subjects who satisfy all criteria will be assigned a random number using opaque sealed envelopes. The random number sequence is generated by SAS ver.9.0 for Microsoft Windows (SAS Institute Inc, Cary, NC) in a 1:1 ratio. The random number generation and the storage of the envelopes will be independently managed by a third researcher. Neither assessors nor subjects will know the assigned group and the random assignment shall not be known to both until the study is terminated. For blinding, the practitioner should perform only the treatment session and minimize the conversation with subjects. The assessors will perform evaluations independently.

### 2.4 Interventions

Enrolled subjects will receive total of 2 treatment sessions of acupuncture. Time points and details of treatment are as shown in Table 1. Information about the acupuncture treatment details based on the Standards for Reporting Interventions in Clinical Trials of Acupuncture is described in Table 2. After the completion of each treatment session, the experimental group and the control group will be evaluated for the position of the acupuncture using the PF-X-ray and the accuracy of the acupuncture will be evaluated as a success or failure.

**Table 1:**
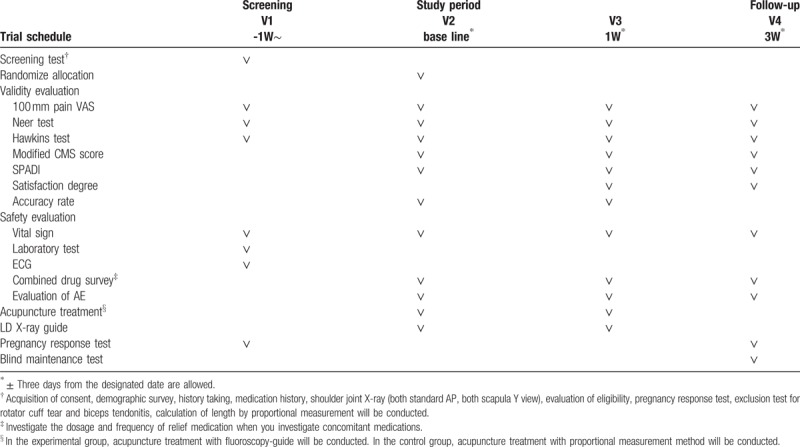
Progress table of clinical trial.

**Table 2:**
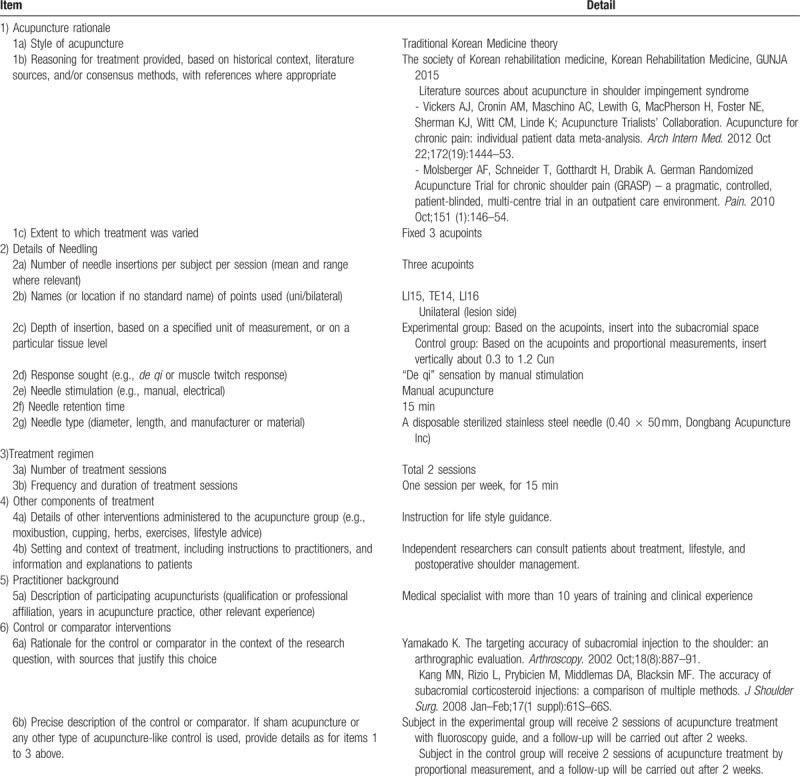
STRICTA.

#### 2.4.1 Experimental group

Subjects in the experimental group will receive acupuncture treatments to the subacromial space with a fluoroscopy guide. The acupuncture points will be LI15, TE14, and LI16 on the side of the affected shoulder. Acupuncture treatment will be performed for 15 minutes with needle manipulations. We will use disposable sterile acupuncture (0.40 × 50 mm, Dongbang, Republic of Korea). For the acupuncture guide, we will use the PF-X-ray. Before the acupuncture needles are inserted, the shoulder anteriorposterior (AP) view is taken in radiography mode to confirm the subacromial space (however, it may be omitted depending on the patient's condition). We will perform acupuncture treatments with a PF-X-ray guide in fluoroscopy mode.

#### 2.4.2 Control group

Subjects in the control group will receive acupuncture treatments with proportional measurements not using fluoroscopy. Acupuncture points are same as those for experimental group and the locations and depths of acupuncture points will be according to the Korean Medicine Convergence Research Information Center (KMCRIC) standard acupuncture point guideline and proportional measurements.

### 2.5 Outcomes

#### 2.5.1 Primary outcome

The primary outcome will be the change 100 mm pain VAS for shoulder pain. For rating severity of shoulder pain intensity, 100 mm pain VAS will be assessed by the subjects self-checking and evaluating the degree of pain using the 100 mm line, which is described at one end as “no pain” and at the other end as “worst pain imaginable.” This outcome will be measured at visits 2, 3, and 4. At each measurement, the shoulder pain intensity that occurs during the Neer Test and the Hawkins Test will be measured.^[11]^

#### 2.5.2 Secondary outcomes

The secondary outcomes will be the tests for impingement sign and function of the shoulder joint. These will be measured at visits 2, 3, and 4. To assess improvement of impingement syndrome, we will perform the Neer test and Hawkins test.^[11]^ Function of the shoulder joint will be assessed by the Modified Constant Murley Score scale (Modified CMS) and the Shoulder Pain And Disability Index (SPADI). We will also evaluate the patient satisfaction degree and accuracy rate of acupuncture. Using the PF-X-ray, we will check the shoulder standard AP view and scapular Y view. We will rate the procedure as a success when the acupuncture needle is accurately inserted into the subacromial space.

#### 2.5.3 Safety evaluation

We will check the vital sign at every visit. We will check for the adverse events (AEs) and serious adverse events (SAEs) at every visit of the study. For AEs and SAEs, we will record the onset date, end date, the intensity, the relationship with the clinical trial, the result, and the action taken to address the AE. When the SAE occurs, we will take an appropriate measure to minimize adverse reactions and report to the IRB within the period set by the IRB.

### 2.6 Sample size

For calculating the sample size, we searched for a clinically significant difference of VAS in previous studies. There was no previous study with the same experimental and control groups as ours evaluating the efficacy with the VAS as a primary outcome. So referring to the previous clinical trial, which included a broad population of patients with various musculoskeletal conditions, we determined a significant difference of VAS as 2 point.^[12–14]^ From the study comparing fluoroscopically guided and blind corticosteroid injections, standard deviation was conservatively assumed to be 2.3.^[15]^ We calculated the sample size with 80% power at a significance level of 0.05. The sample size is 42 in total and 21 for each group. Assuming a dropout of about 20%, about 54 patients in total (27 per each group) may be needed.

### 2.7 Statistical analysis

For statistical analysis, a full analysis set (FAS), per-protocol set (PPS), and the safety set will be used. Both the FAS analysis and the PPS analysis will be used for the efficacy evaluation. PPS will be the main set according to the principle of intentionality treatment.

Evaluation of the primary outcome will be conducted by VAS change after 2 treatment sessions (visit 3) compared with baseline (visit 2). To compare 2 groups, 2-sample *t* test will be used. The paired *t* test will be used for analysis in each group. For the interaction of treatment over time, repeated measures analysis of variance will be used. And secondary outcomes (modified CMS and SPADI) are evaluated by the same methods as the primary outcome. The Neer test and Hawkins test will be summarized as frequency and percentage, and the comparison between the 2 groups will be performed using the *χ*^2^ test or the Fisher exact test. The accuracy ratio acupuncture is calculated by comparing the ratio between the 2 groups.

For safety evaluation, a 2-sample *t* test or Mann–Whitney *U* test will be conducted in case of continuous variable, and *χ*^2^ test or Fisher exact test will be conducted for assessing the differences of incidence of adverse events.

In the case of missing data, the method of last observation carried forward (LOCF) will be performed.

### 2.8 Data management

All trial data will be recorded in accordance with the standard operating procedure of the Wonkwang Gwangju Hospital clinical trial center (WCTC). And all data and source documents will be archived with full backups in the WCTC computer system.

## 3 Discussion

Shoulder pain is one of the common musculoskeletal disorders that can reduce the quality of life, and SIS is one of the major causes of it. To treat SIS, corticosteroid injection therapy is commonly used to reduce inflammation and pain in the subacromial space. However, steroid injection can cause the weakening of the collagen fiber and it can bring about the rotator cuff tear.^[16]^ Thus, it is used only when conservative treatments, such as nonsteroidal anti-inflammatory drugs, exercise, and physical therapies have failed.

Acupuncture has traditionally been performed for treatment of chronic pain and is also effective in treating SIS.^[17]^ For SIS treatment, acupuncture is usually performed on the acupoints located on the shoulder including the subacromial joint space. According to the classical acupuncture theory, the location of acupoints is decided by the Cun measurement system, which measures the length according to the proportional method based on palpation. Cun is the unit used to measure the length in classical acupuncture proportional measurement. The distance from the cubital crease to the palmar wrist crease is calculated as 12 Cuns. So we measure the length of 1 Cun and apply acupuncture treatment according to the KMCRIC standard guideline.^[18,19]^

According to meta analyses about the effectiveness of steroid injection in SIS, it is reported that the effect of steroid injection is controversial.^[6,20,21]^ And the effectiveness can be affected by the accuracy of the injection technique.^[10,22]^

Acupuncture treatment in the accurate subacromial joint space may have a therapeutic advantage over the injection. However, it is very difficult to apply acupuncture treatment accurately using only the proportional measurement and palpation guide. Furthermore, there was no study of the accuracy rate of acupuncture on SIS. This protocol is intended to evaluate the effectiveness and safety of fluoroscopy-guided acupuncture for SIS using PF-X-ray. In addition, we can evaluate the accuracy rate of acupuncture treatment compared with the conventional proportional measurement method. A clinical trial with this protocol can provide a new perspective on the practice of acupuncture with a fluoroscopy-guide.

## Author contributions

JS: Conception and design of the trial, wrote protocol, drafted manuscript

S-RY: Wrote protocol, drafted manuscript, conception and design of the trial, data collection and analysis, interpretation

H-RS: Wrote protocol, drafted manuscript, data collection and analysis, interpretation

KP: Data collection and analysis, interpretation, critically revised the protocol

JKK: Wrote protocol, data collection and analysis, critically revised the protocol

S-JP: Data collection and analysis, interpretation, critically revised the protocol

SL: Wrote protocol, drafted manuscript, conception and design of the trial, critically revised the protocol

**Investigation:** Hee-Ra Shin, Kyungtae Park, Soo-Ji Park.

**Project administration:** Seung-Ryong Yeom.

**Supervision:** Sangkwan Lee.

**Writing – original draft:** Jihye Seo.

**Writing – review & editing:** Seung-Ryong Yeom, Hee-Ra Shin, Jae Kyoun Kim, Sangkwan Lee.
